# Indirect surgical revascularization for management of vascular steal phenomenon in high-grade untreatable brain arteriovenous malformations

**DOI:** 10.1007/s00381-025-06844-y

**Published:** 2025-06-09

**Authors:** Vitor Nagai Yamaki, Sanjay Bhate, Vijeya Ganesan, Fergus Robertson, Adam Rennie, Greg James, Adikarige Haritha Dulanka Silva

**Affiliations:** 1 Department of Neurosurgery, Great Ormond Street Hospital for Children, NHS Foundation Trust, London, UK; 2 Department of Neurology, Great Ormond Street Hospital for Children NHS Foundation Trust, London, UK; 3 Department of Interventional Neuroradiology, Great Ormond Street Hospital for Children, NHS Foundation Trust, London, UK

**Keywords:** Brain arteriovenous malformation, Cerebral revascularization, Transient ischaemic attacks

## Abstract

**Purpose:**

To discuss the role of surgical revascularization (SR) in inoperable deep-seated brain arteriovenous malformation (bAVM) to treat ischaemic symptoms owing to cerebral hypoperfusion from vascular steal in paediatric patients with vascular steal phenomenon.

**Methods:**

We discuss the management of two children with high-grade deep-seated bAVMs who underwent indirect SR with encephalo-duro-arterio-myo-synangiosis (EDAMS) and superficial temporal artery (STA) pial synangiosis. Long-term clinical and radiological follow-up were presented.

**Results:**

Both children aged 11 and 14 years with deep-seated basal ganglia/thalamic bAVMs (Spetzler Martin 5, Lawton-Young Supplemented Score 8 who presented with ischaemic-related symptoms (progressive motor deficit). There were no ischaemic lesions evidenced on the MRI. EDAMS with STA pial synangiosis was performed with marked improvement of the progressive and fluctuating symptoms. At 2-year follow-up, angiogram showed satisfactory revascularization from the STA graft to the hypoxemic peri-Rolandic territories. Both patients presented stabilization of clinical symptoms up to 4-year follow-up after IR. There was no evidence of haemorrhage, ischaemia or bAVM progression on follow-up imaging.

**Conclusion:**

SR using indirect techniques may have a role in the management of inoperable deep-seated bAVMs with symptoms due to vascular steal phenomena. The successful revascularization of these cases may also provide evidence to support the hypothesis of vascular steal phenomenon in rapid shunting bAVM causing a local perinidal hypoxemia and neural damage.

## Introduction

High-grade brain arteriovenous malformations (bAVMs) pose a significant management challenge owing to the lack of definitive therapeutic interventions for directly treating the bAVM. In many cases, such bAVMs cannot be cured: partial treatment of bAVMs has yet to be shown to alter natural history of these lesions and may result in poorer outcomes [1]. Therapeutic efforts are therefore aimed at modifying specific morphological or flow-related features of the lesion to reduce future risk of haemorrhage (if high-risk features such as flow aneurysms are present).

Patients with high-grade bAVMs can present with non-haemorrhagic neurological deficits [1]. The attribution of this clinical semiology to ‘vascular steal’ phenomenon is controversial [1–3] but may be mediated by factors including size, angioarchitecture and flow characteristics of the bAVM [1]. Whilst studies have demonstrated that there was no evidence of reduced cortical cerebral blood flow (CBF) [4], in the presence of high-flow bAVM, there may be local disturbances of cerebral haemodynamics [5]. A specific group of patients may present with fluctuating symptoms suggestive of worsening neurological deficits that may not correlate to the anatomical location of the bAVM itself and with little or no ischaemic findings on magnetic resonance imaging (MRI) [1, 6].

The is no consensus on strategies to treat ‘ischaemic’ type semiology in this cohort. The role of surgical revascularization (SR) has not been well documented aside of a single reported case in the paediatric literature reported with short term follow-up [1].

SR utilizing a variety of techniques to augment cerebral blood flow to hypo-perfused brain [5] is well established in the context of children with conditions of impaired cerebral perfusion, the most common of which is moyamoya disease (MMD) [7–9]. The distribution of hypoxemic regions in the cerebral hemisphere can be established in perfusion studies as well as radiological evidence of previous arterial ischaemic strokes (with corresponding encephalomalacia and volume loss) and by the recently described ‘Ivy’ sign which represents a slow or stagnant cortical blood flow in the region of hypoperfusion [10] and may demonstrate radiographic and clinical utility as a prognostic biomarker postoperatively following SR. Impaired cerebral perfusion in the context of cerebral proliferative angiopathy (CPA) has also been documented, and in selected patients and circumstances, SR with indirect techniques has been employed as a successful management strategy in the context of presentation with ischaemic symptoms [11–13].

We present the cases of two children with large, high-grade bAVMs presenting with clinical and radiological features of vascular steal with semiology akin to that of cerebral hypoperfusion and cerebral ischaemia managed with indirect SR utilizing pial synangiosis with superficial temporal artery (STA) graft with the aim of alleviating and stabilizing the symptomatology and attempt to possibly reduce the risk of future ischaemic events.

### Case reports

#### Case report 1

Fourteen-year-old male who presented with a 10-month history of progressive left-sided hemiparesis, exacerbated by exertion, hot weather and dehydration. There was a family history of vascular pathology with his mother having a recent diagnosis of brain aneurysm, maternal grandfather with a ruptured brain aneurysm and maternal first cousin with a bAVM. He was referred from another institution following detailed neurological assessment.

He was screened for mutations in a neurovascular malformations panel. Hereditary Haemorrhagic Telangiectasia (HHT), Activin A Receptor Like Type 1 (ACVRL1), Mothers Against Decapentaplegic homolog 4 (SMAD4), Phosphatase and Tensin homolog (PTEN), Ras p21 protein activator 1 (RASA1), PIK3 CA and Ephrin 4 (EPHB4) testing were all negative for pathogenic mutation.

**Fig. 1:**
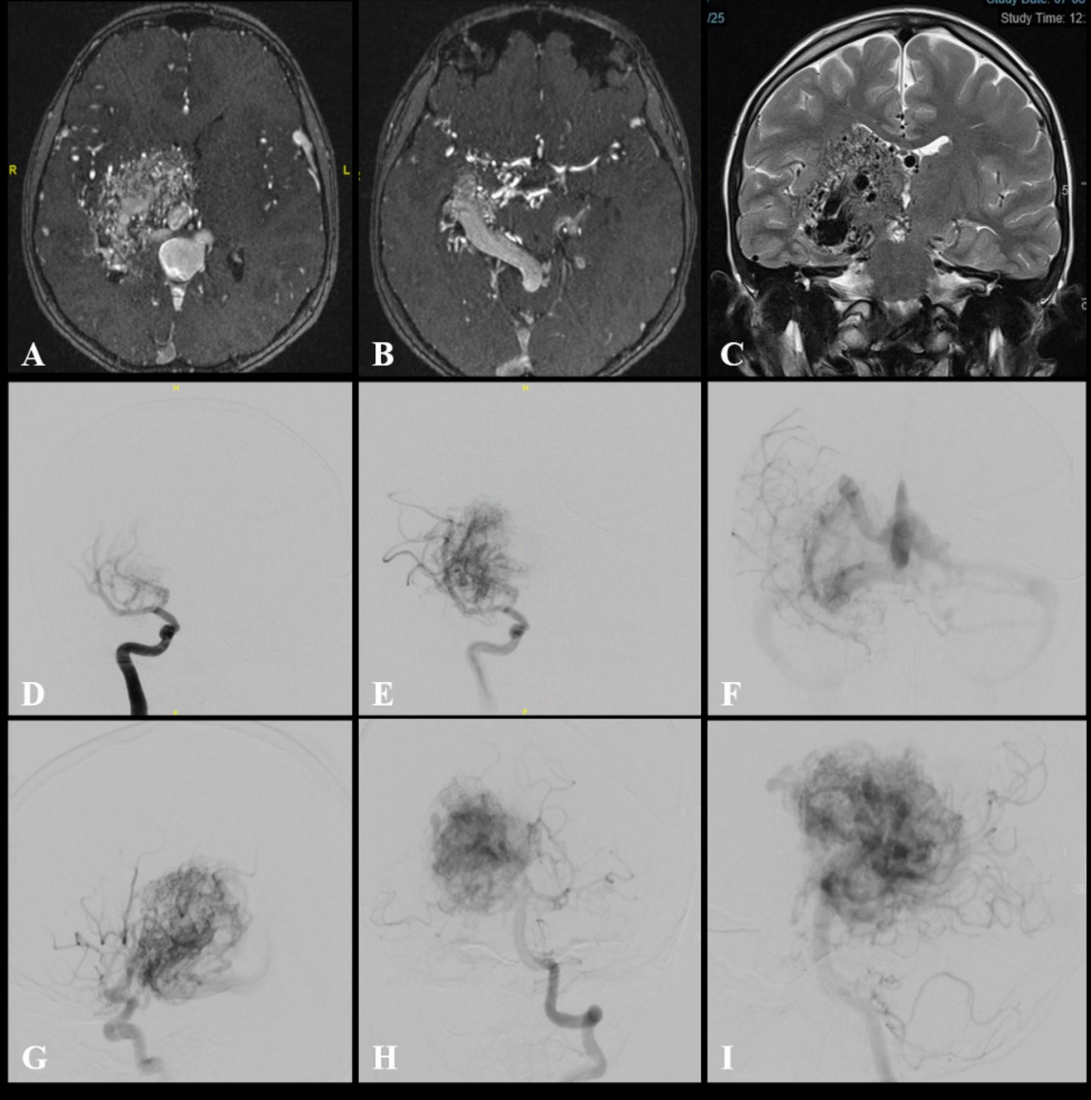
**A**, **B** Axial MR angiography (MRA) views of the right-sided deep-seated bAVM centred on the right thalamus. **B** The AVM has arterial feeders from anterior and posterior circulations and deep venous drainage towards the vein of Galen where it is associated with a dilated venous pouch. **C** Coronal T2-weighted image demonstrating the bAVM flow-voids at the right thalamic region extending to the basal ganglia where it is supplied by the lenticulo-striate and choroidal perforating arteries. Note there is no evidence of direct encephalomalacia changes and injury within the subcortical structures **D**–**F** anterior–posterior (AP) digital subtraction angiography (DSA) of the right internal carotid artery (ICA) injection confirming multiple arterial supply from ACA A1 and MCA M1 lenticulo-striate arteries and relatively rapid shunting with **F** early venous deep drainage. **G** Lateral DSA demonstrating early feeling of the bAVM nidus supplied by the anterior choroidal artery. **H**, **I** AP and lateral DSA of the left vertebral artery (VA) depicting the contribution of the posterior circulation to the arterial supply of the bAVM from the posterior cerebral artery (PCA) P1 segment (P1), posterior communicating artery (PCommA), and posterior choroidal artery (PChA). There is no supply to the bAVM from the hemispheric cortical vasculature of the anterior or posterior circulations. There were no radiological features to suggest a diagnosis of cerebral proliferative angiography (CPA) either. Notice the paucity and slowness of the hemispheric cortical vessel filling as a result of the degree of high-flow shunting through the bAVM

Cerebral angiography confirmed the presence of a right thalamic/choroidal bAVM with anterior and posterior circulation supply by multiple arterial feeders from perforating vessels: anterior cerebral artery (ACA) A1 and middle cerebral artery (MCA) M1 leticulo-striate arteries, posterior cerebral artery (PCA) P1 and posterior communicating arteries (PCommA) and from the anterior (AChA) and posterior choroidal (PChA) arteries. There was deep venous drainage towards the dilated vein of Galen. This was classified as Spetzler-Martin grade 5 and Lawton-Young supplemented score 8 (SM5 LY8). There was no identified external carotid arterial supply, no obvious aneurysms or varices and no trans-dural collaterals (Fig. 1). The angiographic features were not felt to be representative of CPA. There was no supply to the bAVM from the hemispheric distal cortical vasculature.

**Fig. 2:**
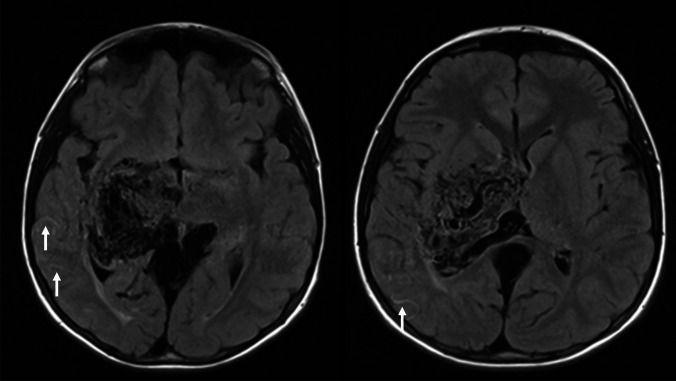
MRI FLAIR axial sequences demonstrating evidence of Ivy sign in the cortical sulci in the right posterior temporal regions. There is no evidence of direct injury or encephalomalacic changes within the subcortical structures

The child was initially managed with optimization from a haemodynamic perspective – advice of adequate hydration on a daily basis with increased intake advised during exertion, concurrent illness and hot days, akin to management for patients with MMD – but over a period of a year and a half developed progressively worsening hemiparesis with progression of the left upper limb weakness and a visual field defect with left side hemianopia. Repeated MRI and MR angiography (MRA) showed stable appearances with no progression or radiological changes in the bAVM. However, there was evidence of Ivy sign in the right hemisphere on the new FLAIR MRI sequences suggesting a degree of stagnant cortical flow without obvious ischaemic lesions (Fig. 2). He progressed in terms of symptomatology to needing a wheelchair over the subsequent months for mobility.

The symptomatology was felt to be attributable to progressive vascular steal due to the deep-seated high-grade bAVM. The case was discussed within our local neurovascular multidisciplinary team board (MDT) meetings and with international colleagues to consider treatment options to manage the bAVM. Options of partial endovascular embolization, Gamma Knife or Cyber Knife stereotactic radiosurgery (SRS) were discussed, but it was felt that the size of the lesion would carry a significant risk of morbidity and therefore was excluded.

**Fig. 3:**
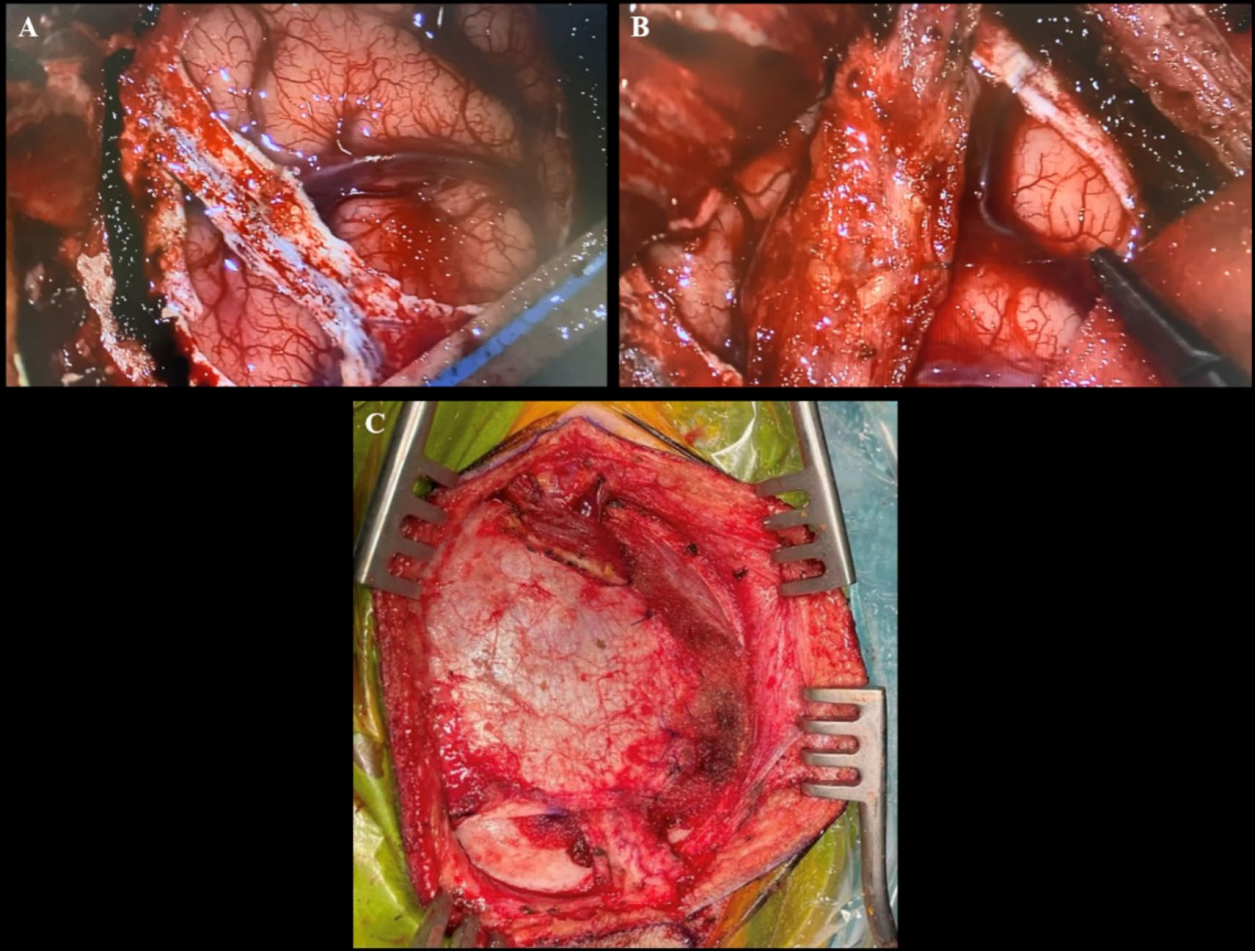
**A** Intraoperative view of the dural opening preserving the arterial supply from the middle meningeal vessels on the cut edges of dura. A large arterialized and dilated superficial sylvian vein is visualized. The brain was hyperdynamic as a result of the hyperdynamic circulation. **B** Pial synangiosis utilizing superficial temporal artery (STA) with an adventitial cuff with apposition to the pial surface of the MCA territory with 9/0 prolene sutures. **C** Final view of the encephalo-duro-arterio-myo-synangiosis (EDAMS) with temporalis and muscle fascia utilized for closure of the dural defect over the STA arterial graft. The postoperative DSA at 1 year following SR demonstrated evidence of revascularization via the arterial graft particularly in the peri-Rolandic region (Fig. 4). On the last clinical follow-up at 2 years following SR, the left-sided hemiparesis has stabilized. He has not had any further ischaemic symptoms and is now no longer utilizing a wheelchair but is able to walk independently with ankle–foot orthosis for the left lower limb

**Fig. 4:**
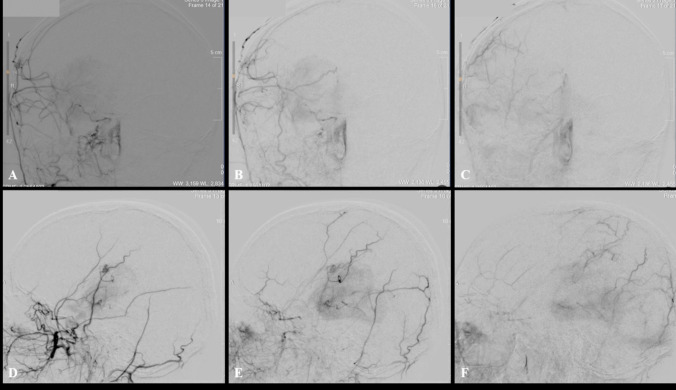
DSA at 1 year following surgery. **A**–**C** AP and **D**–**F** lateral views of the right external carotid artery (ECA) injection showing a degree of cortical augmentation of the peri-Rolandic MCA. There has been no obvious radiological recruitment to the bAVM nor change in its morphology

Given the clinical and radiological presence of features of cerebral hemispheric hypoperfusion, the role of indirect SR was considered. This was discussed with the patient and family and proposed as a possible surgical option for stabilizing and potentially alleviating the disabling symptomatology, and informed consent was obtained. We performed an indirect SR utilizing our previous published technique [7] of encephalo-duro-arterio-myo-synangiosis (EDAMS) with superficial temporal artery pial synangiosis over the right middle cerebral artery (MCA) territory (Fig. 3). There were no perioperative complications, and the patient had an uneventful postoperative course with satisfactory revascularization to the cerebral hemisphere (Fig. 4). At the 2-year follow-up, the left-sided weakness had improved (although not completely resolved), and exacerbations previously triggered by dehydration or hot weather were no longer observed. In addition, he improved to mobilization with at first review at 1 year, with a single stick and then at 2 years to mobilizing independently.

#### Case report 2

Eleven-year-old girl, who first presented clinically with a progressive right-sided weakness, hypoesthesia affecting the upper, lower limbs and face and intermittent headaches. She also had a significant degree of fatigue and problems with concentration at school which was impairing function and necessitating time off school. She had a background history of epistaxis during infancy and was reported to have had a previous old MRI which had been reported as ‘a small vascular lesion in the left thalamus’ at an outside institution. These images were not available for our review, and she had not been followed up at the time. A clinical assessment felt that these episodes were vascular events rather than seizures, and a formal EEG study captured no events with no focal or epileptiform abnormalities observed.

The genetic screening for HHT and RASA1 mutations was negative.

**Fig. 5:**
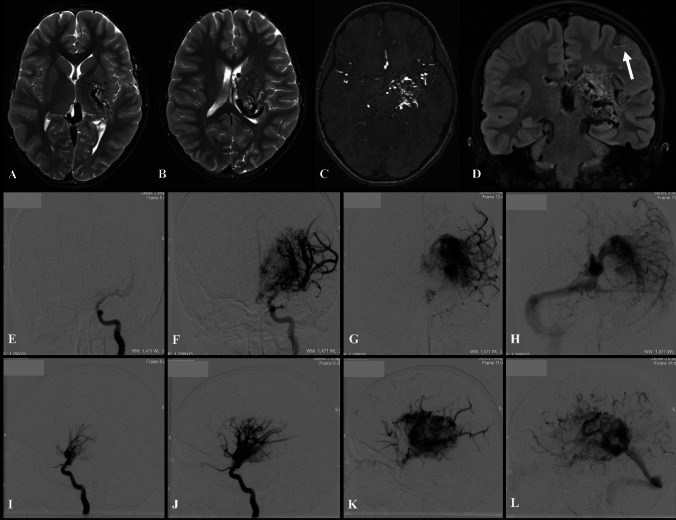
**A**, **B** T2-weighted MRI showing a left deep-seated thalamic/basal ganglia bAVM with deep venous drainage through left thalamo-striate vein towards the vein of Galen. Note the subtle prominence of cortical sulci and cerebrospinal fluid (CSF) spaces on the left frontal and parietal regions compared to the right hemisphere consistent with a subtle degree of volume loss. There is no direct encephalomalacic changes within the subcortical structures to suggest direct injury to the region. **C** MRA showing nidal component on the left thalamic/basal ganglia bAVM with supply from perforating vessels. **D** Coronal FLAIR MRI sequence showing Ivy sign in the left peri-Rolandic sulci. (arrows). **E**–**H** AP view of DSA after left ICA injection showing rapid shunting of the AVM nidus and early venous drainage. **E**, **F** There is an arterial supply predominantly from MCA branches (left lenticulo-striatal, Heubner) and anterior choroidal artery. **I**–**L** Lateral view of the DSA evidencing the AVM supply by the anterior choroidal (**I**) and lenticulo-striatal vessels (**J**). Notice also the paucity and slow filling of the cortical hemispheric vasculature as a result of the high-flow and rapid bAVM shunting

An updated MRI scan for the symptomatology demonstrated a left thalamic/basal ganglia bAVM with Ivy sign affecting the left frontal and parietal hemispheric regions centred in the peri-Rolandic regions with subtle asymmetry and reduction in the cortical volume on the left compared to right hemisphere (Fig. 5). On DSA, there was evidenced a left deep-seated thalamic/basal ganglia AVM (Spetzler-Martin grade 5; and Lawton-Young score 8), supplied by the anterior and posterior circulations by the left lenticulo-striatal, Heubner, anterior and posterior choroidal arteries. A relatively rapid shunting was depicted on DSA with deep venous drainage through a dilated left thalamo-striate vein towards vein of Galen (Fig. 5). The hemispheric cortical vessels demonstrated a paucity of filling due to the rapid degree of bAVM shunting. Again, there was no obvious volume loss in the thalamic or basal ganglia regions to suggest a direct subcortical injury to these structures secondary to the bAVM. There was no obvious hemispheric cortical vascular supply to this bAVM, and the supply was predominantly from the deep-seated lenticulo-striate and choroidal supply. There was no external carotid supply to this bAVM nor evidence of trans-dural collaterals. There were no features to suggest a diagnosis of CPA.

**Fig. 6:**
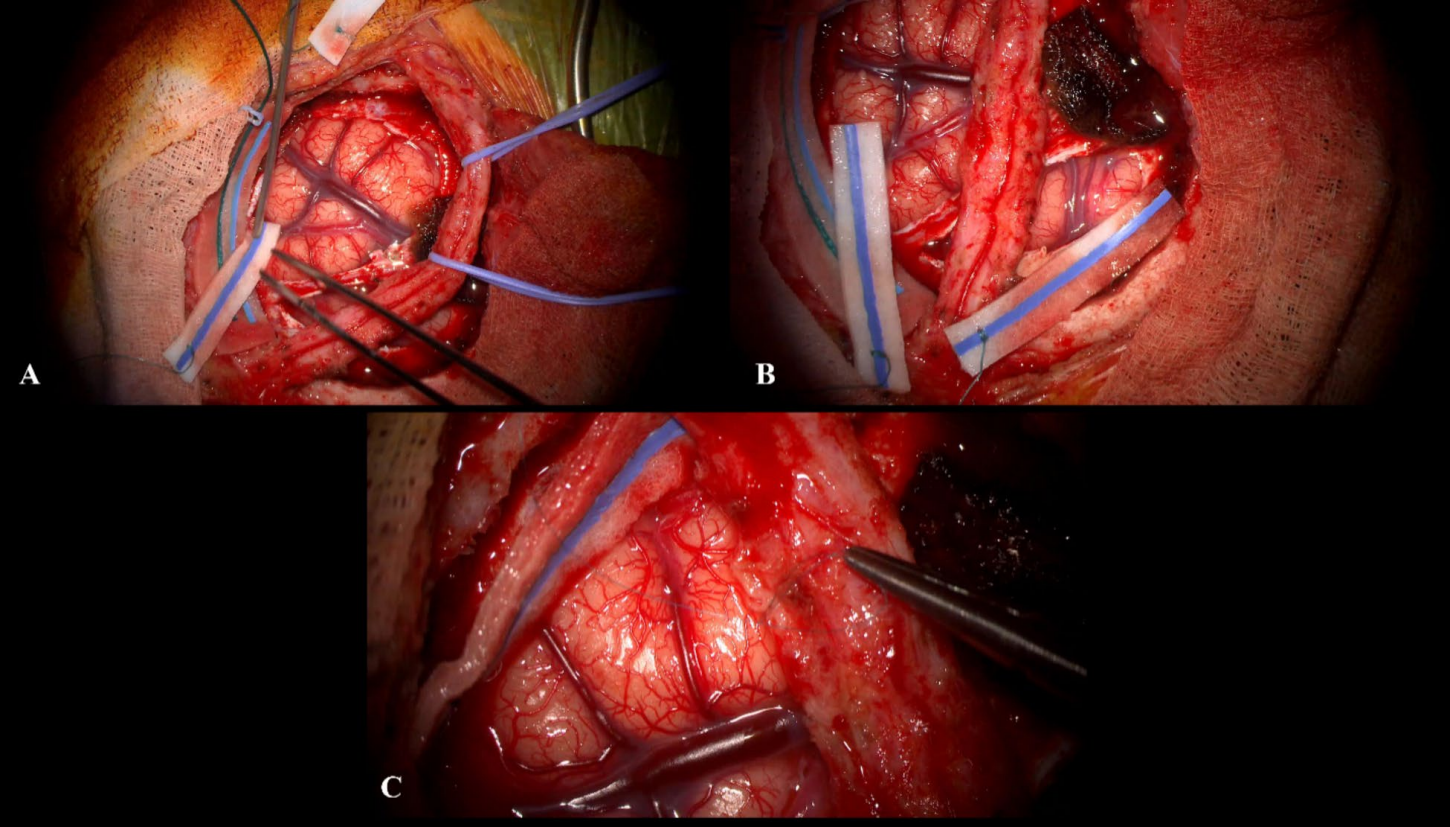
**A**, **B** Exposure with meningeal vessels skeletonized and dural flaps inverted underneath the craniotomy edges to provide a further source of revascularization. Superficial temporal artery (STA) graft with adventitial cuff retracted with vessels loupes. A partially arterialized superficial sylvian vein is visualized. **C** Pial synangiosis with 9/0 prolene to the STA graft and perivascular adventitial cuff to peri-Rolandic pial surface

The symptoms were felt to be attributable to progressive vascular steal due to the deep-seated high-grade bAVM, and, much akin to our first patient, after discussion within the team and international colleagues and after consideration of other therapeutic options directly targeting the bAVM, the role of indirect SR was considered. This was discussed with the patient and family and proposed as a possible surgical option for stabilizing and potentially alleviating the disabling symptomatology, and informed consent was obtained. An identical indirect SR with encephalo-duro-arterio-myo-synangiosis (EDAMS) with superficial temporal artery pial synangiosis over the left middle cerebral artery (MCA) territory (Fig. 6) was performed. There were no perioperative complications, and the patient had an uneventful postoperative course.

**Fig. 7:**
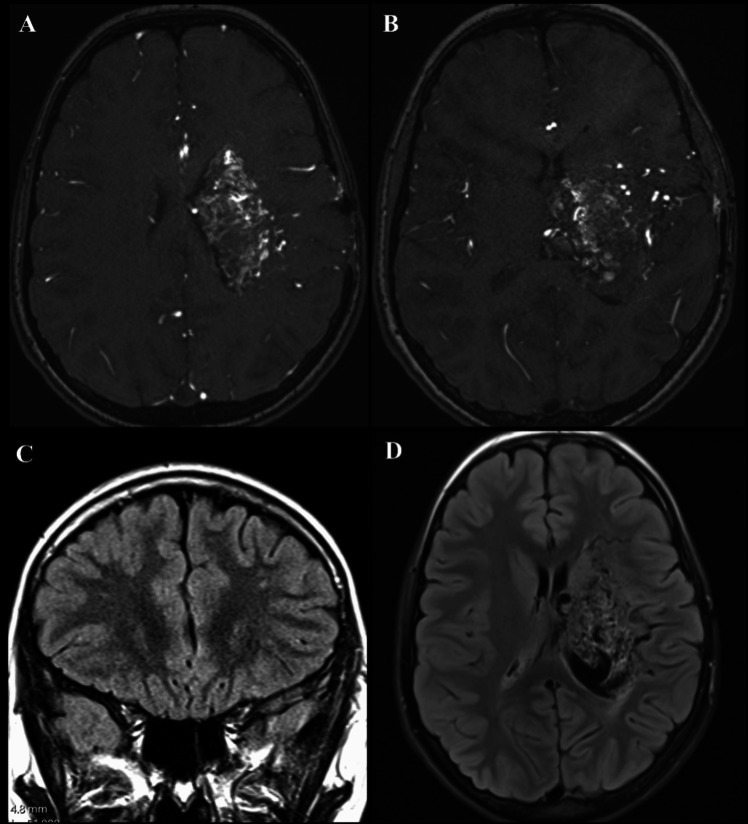
**A**, **B** Postoperative MRI at 6 months showing stable bAVM appearances and new cortical supply from the STA graft over the cortical surface. **C**, **D** Postoperative FLAIR MRI sequence showing reduction in the Ivy sign within the sulci

The patient developed significant improvement with stabilization of her ischaemic symptomatology. There was an improvement in her fatigue and concentration and reduction in headache by 6 months, and she was returning back to school as usual. The 6-month postoperative MRI showed improvement of the Ivy sign, no ischaemic lesions and no obvious structural changes of the AVM (Fig. 7).

**Fig. 8:**
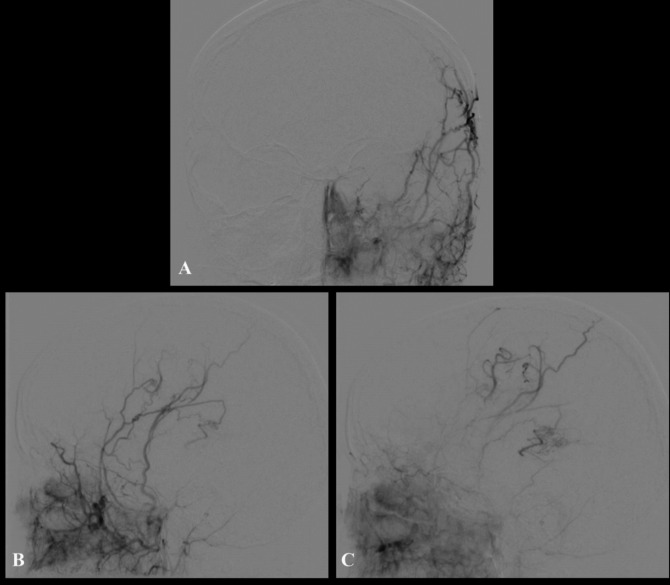
**A** AP view of postoperative DSA of ECA injection demonstrating collateralization and hemispheric augmentation through the synangiosis graft. **B**, **C** Lateral view of postoperative DSA of ECA injection demonstrating revascularization and collateralization both via the STA synangiosis graft as well as dural vessel-related collaterals over the exposed peri-Rolandic regions

A further DSA was performed at 14 months following surgery which demonstrated no morphological changes to the bAVM. There was excellent collateralization and revascularization via both the STA synangiosis graft and via dural meningeal vessels to the peri-Rolandic region consistent with successful revascularization and hemispheric augmentation (Fig. 8). On the last clinical follow-up 2 years after revascularization surgery, there were no further clinical deterioration and complete stabilization of her symptomatology with improvement in her right-sided weakness and hypoaesthesia.

## Discussion

We present the management of two children presenting with deep-seated bAVMs and clinical presentation commensurate with cerebral hypoperfusion and progressive ischaemic symptomatology – somewhat homologous to that observed in MMD and ischaemic CPA. Clinical semiology, responses to hydration and radiological features corroborated the hypothesis of cerebral hypoperfusion and likely vascular steal phenomenon, and after much consideration, we elected to treat these patients with a similar paradigm utilizing indirect SR [14, 15]. To our knowledge, this is the first time in the literature indirect SR has been performed to provide hemispheric augmentation in deep-seated bAVMs with vascular steal in children and adults. There is evidence of the role of SR in ischaemic CPA in adults and children.

The concept of vascular steal phenomenon in bAVM states that the blood flow is diverted towards a low resistance vessel network into the AVM promoting a hypoperfusion of the surrounding brain [3, 16]. Even though reliable haemodynamic models have demonstrated flow changes in the bAVM, it is still controversial whether it impacts brain perfusion due to an adaptive autoregulatory capillary recruitment [17]. Anglani et al. [18] demonstrated a diffuse hypometabolism on the ipsilateral hemisphere of the AVM on perfusion study with ^18^F-fluorodeoxyglucose positron emission tomography-magnetic resonance imaging (FDP-PET MRI). After partial embolization of the high-flow feeders, there was documented a significant improved of the cortical hemispheric peri-lesional hypometabolism.

In considering the role of revascularization for hemispheric augmentation of flow, imaging evaluation of cerebral blood flow with perfusion imaging such PET or SPECT may be considered to confirm cerebral hypoperfusion. In our institution, we do not routinely utilize perfusion imaging in the diagnostic or management paradigm for patients with occlusive arteriopathy being considered for cerebral revascularization owing to the additional constraints implicit in a paediatric population [19]. The routine implementation of perfusion imaging in the management of occlusive arteriopathy in children is however variable in different institutions and not routinely employed. In their landmark paper on long-term outcomes of pial synangiosis in children with moyamoya, Scott et al. [20] do not routinely utilize perfusion imaging in their management paradigm. The role of techniques to measure cerebral perfusion in the diagnosis and follow-up of children with moyamoya was felt to be a Class IIb recommendation with level of evidence C and not imperative but a useful adjunct [21]. The recent European Stroke Organisation (ESO) guidelines on moyamoya angiopathy [22] also found on analysis of current evidence that there were no randomized control trials and no comparative studies specifically evaluating the effectiveness of the assessment versus no assessment of haemodynamic status in patients with moyamoya angiopathy. Ellis and colleagues also did not utilize perfusion imaging in the decision-making or management paradigm for their patient with vascular steal phenomenon managed with pial synangiosis [1]. We do acknowledge however that as the efficacy of revascularization as a treatment option in this circumstance is not proven in the literature, as part of a comprehensive preoperative evaluation, perfusion imaging would be beneficial.

In deep-seated bAVMs supplied primarily by subcortical perforating vessels, a degree of steal can be hypothesized as being related to the degree of high-flow shunting through the bAVM with a paucity of filling and slowness of filling in the distal hemispheric vasculature. In both our patients, there were a paucity and slowness of filling of the hemispheric cortical vasculature on DSA, and on MR imaging, there were radiological features suggestive of hypoperfusion with the presence of Ivy sign, the latter being well substantiated in terms of its role as a radiographic diagnostic and prognostic biomarker for brain perfusion in paediatric moyamoya that may potentially subserve to eliminate radiation exposure and additional time and cost associated with CT perfusion with acetazolamide challenge [10].

After cerebral revascularization, fluctuating and progressive symptoms have improved in both patients with stabilization of the neurological deficits. Again, we do accept that a pre- and postoperative evaluation of blood flow using PET or SPECT would have been useful to provide further objective radiological evidence supporting the clinical improvement observed in patients. There were no structural or morphological changes to the bAVMs at last follow-up. The postoperative DSA demonstrated a good degree of revascularization and collateralization to the peri-Rolandic regions via the STA pial synangiosis graft and via the dural meningeal vessels bypass without obvious recruitment of the bAVM. As well as providing indirect evidence to support the presence of vascular steal phenomena in the context of high-flow bAVMs, our revascularization surgery in these two children may also suggest a role for hemispheric cerebrovascular augmentation utilizing external carotid artery sources in carefully selected patients with ischaemic semiology owing to vascular steal from deep-seated bAVMs.

Ischaemic symptoms have been described in a specific group of patients harbouring a large, diffuse lesion with multiple small arterial feeders, deep venous drainage and a puddling appearance in the venous phase of the angiogram with no high-flow fistulous components [6, 23]. These lesions were named as CPA with a specific natural history which differs from bAVM [6]. Ellis et al. [1] suggested that those large deep-seated AVMs might have similar behaviour as CPAs, and their paper demonstrates the utilization of indirect SR to help stabilize such ischaemic symptoms; however, on the presented DSA, the radiological appearances of their patient were more akin to that of CPA-like appearances as stated by the authors.

The two patients presented had typical radiological appearances of high-flow bAVM, well defined nidus with a relatively rapid shunting not consistent with that of CPA, although this differential was considered.

Revascularization surgery has been shown as a potential treatment for management of ischaemic-like symptoms of CPA; however, there is still incipient evidence regarding long-term outcomes [13, 15]. The rational of providing additional blood supply to the perinidal oligaemia to attenuate CPA angiogenic progression has been postulated, in addition to the clinical evidence of improvement of the ischaemic-related symptoms [24].

Some concerns regarding revascularization surgery for bAVMs rely on the theoretical risk of promoting angiogenic changes and an increased risk of rupture. bAVMs in children exhibit specific behaviour with augmented angiogenic potential compared to adults [25]. Several angiogenic pathways are influenced by pathways of aging and senescence [25, 26]. The vascular endothelial growth factor (VEGF) is the main pathway for initiation and progression of bAVM [27]. It is initially triggered by hypoxia-induced factor 1α (HIF-1α), endothelial nitric synthase and metalloproteinases induced by the perinidal hypoxemic environment [28, 29].

The possible recruitment of revascularization graft into the vascularity for the bAVM and its potential to alter the natural history of the bAVM was considered. It is important to ensure cortical arteries are not feeder vessels to the AVM. Taking the analogy of revascularization surgery in the context of cerebral proliferative angiopathy (CPA), another vascular malformation that lies in the spectrum of arteriovenous malformations in general, there is described in the literature a case where revascularization has been performed for CPA and there is recruitment of the vascularized graft into the feeder vessels to the CPA with associated early venous shunting [30]. In our analysis of the bAVM, it was felt to be predominantly supplied from perforating vessels with no supply from cortical hemispheric vessels. Our surgical aim was therefore to only attempt to augment the superficial hemispheric vessels distal to the bAVM to ameliorate the vascular steal effect. We felt therefore that the lack of a superficial cortical-based bAVM and the presence of these bAVMs being deep-seated and purely subcortical were important factors in our rationale for proceeding with SR. Other factors we considered including the absence of direct subcortical injury to the deep subcortical thalamic/basal ganglia and internal capsule to explain the neurological deficit (i.e. that the symptomatology is truly related to hemispheric vascular steal), the lack of other treatment options for the bAVM with a lower morbidity profile compared to SR and evidence for the cerebral hypoperfusion hypothesis. In our patients, the success of collateralization and revascularization to the hemispheric territory through our STA pial synangiosis graft and the meningeal vessels suggests evidence of a hypoxemic drive in the hemisphere as a result of likely slow flow through the cortical hemispheric vasculature due to the steal from the deep-seated bAVM.

Patient selection is crucial in undertaking these decisions. We feel that multidisciplinary expertise in a high-volume centre with expertise in management of paediatric arteriopathy and revascularization management is crucial to appropriate patient selection.

Possible patient factors and indications to consider revascularization in this cohort may therefore be:

- Presentation with ischaemic semiology consistent with vascular steal and compromise (akin to MMD or moyamoya syndrome)
- Presence of a deep-seated bAVM (thalamic/basal ganglia) with predominantly deep supply and relative hypoperfusion and paucity and slow filling of the hemispheric cortical vasculature as a result of high-flow shunting
- Lack of direct subcortical thalamic/basal ganglia injury in direct relation to the bAVM
- The lack of direct cortical M4 segment supply to the bAVM (to possibly obviate the theoretical risk of recruitment of vascularity to the bAVM directly)
- Evidence of cerebral hypoperfusion, ischaemia and hypoxaemic drive clinically and radiologically (volume loss, ‘Ivy’ sign on FLAIR sequences, previous established arterial infarction, demonstration of cerebral hypoperfusion with cerebral blood flow studies such as PET and SPECT)
- Lack of other treatment options for the bAVM with a lower morbidity profile compared to SR

The indication for indirect revascularization (IR) was driven by progressive neurological deficits during conservative management of both patients. Therefore, a multidisciplinary discussion favouring IR was taken once it was felt that there was no safe surgical, endovascular targets for treatment and the presence of a significantly increased risk of complications and worsening symptoms with stereotactic radiosurgery [31].

Our study has limitations. This is a report of two paediatric patients with a rare presentation of bAVM. We have not performed functional cerebrovascular reserve studies postoperatively following SR. However, as with other established high-volume centres performing paediatric SR [5, 8], we do not routinely perform perfusion studies in our neurovascular practice either pre- or postoperatively relying primarily on clinical symptomatology, radiological and angiographic evidence to demonstrate cerebral hypoperfusion and in essence a ‘hungry brain.’ This has been our practice over nearly three decades [8]. Nonetheless, we do accept that pre- and postoperative cerebral blood flow and perfusion studies in this group of patients would be beneficial in adding further objective evidence to both guiding the decision for revascularization and demonstrating improvement and should be considered. Our follow-up period at present remains 2 years only, and clearly, a longer term careful follow-up of these patients is essential to evaluate and monitor for possible risk of bAVM rupture as well as to ensure that the clinical stabilization remains sustained.

## Conclusion

Progressive ischaemic-like symptoms in bAVMs may be related to vascular steal phenomenon in the perinidal vascular territory and as a result of impairing distal cerebrovascular flow. In carefully selected children with untreatable deep-seated bAVMs presenting with ischaemic symptoms with clinical and radiological evidence of cerebral hypoperfusion, augmentation of cerebral hemisphere perfusion with indirect revascularization techniques may stabilize and alleviate neurological symptomatology and may have a role in the management of these patients.

## Data Availability

No datasets were generated or analysed during the current study.
